# Correction: Verkhovskii et al. The Influence of Magnetic Composite Capsule Structure and Size on Their Trapping Efficiency in the Flow. *Molecules* 2022, *27*, 6073

**DOI:** 10.3390/molecules28155647

**Published:** 2023-07-26

**Authors:** Roman Verkhovskii, Alexey Ermakov, Oleg Grishin, Mikhail A. Makarkin, Ilya Kozhevnikov, Mikhail Makhortov, Anastasiia Kozlova, Samia Salem, Valery Tuchin, Daniil Bratashov

**Affiliations:** 1 Science Medical Center, Saratov State University, 83 Astrakhanskaya Str., 410012 Saratov, Russia; 2 Institute of Molecular Theranostics, I. M. Sechenov First Moscow State Medical University, 8 Trubetskaya Str., 119991 Moscow, Russia; 3 Department of Physics, Faculty of Science, Benha University, Benha 13511, Egypt; 4 Laboratory of Laser Molecular Imaging and Machine Learning, Tomsk State University, 36 Lenin’s Ave., 634050 Tomsk, Russia; 5 Institute of Precision Mechanics and Control, FRC “Saratov Scientific Centre of the Russian Academy of Sciences”, 24 Rabochaya Str., 410028 Saratov, Russia; 6 Bach Institute of Biochemistry, FRC “Fundamentals of Biotechnology of the Russian Academy of Sciences”, 119071 Moscow, Russia

## Text Correction

In the original publication [1], there was a typo in the original publication. **The wrong measurement units were used**.

A correction marked in red has been made to **Results Section, Paragraph 7:**

To evaluate the influence of the viscous drag force of the liquid flow on the magnetic capture efficiency of differently sized capsules, we used the custom-built, SPIM-Fluid based flow cytometer (Figure 2a). Among all sizes of capsules loaded with MNPs by one freezing/thawing cycle, the largest one was found to be the most sensitive to the magnetic field and could be captured at flow velocity up to 100 mm/s. 

A correction marked in red has been made to **Results Section, Paragraph 12**:

Since macrophages are characterized by higher phagocytic activity in comparison with tumor cells. The Raw 264.7 cell line was chosen as a model for the verification of the capability of the magnetic trapping of cells from the flow. Additionally, we chose 2.7 µm capsules loaded by MNPs in three freezing/thawing cycles for these experiments since their internalization efficiency as well as magnetic moment are higher than for 1 µm capsules. The flow velocity was around 5 mm/s to reduce viscous drag force since the size (around 13 µm) and weight of Raw 264.7 cells are much higher than the largest investigated capsules (5.5 µm). 

A correction marked in red has been made to **Discussion Section**, **Paragraph 3**:

According to Ref. [11], 1 µm and 2.7 µm magnetic capsules synthesized from biocompatible polyelectrolytes such as Parg and DS and loaded with MNPs in three freezing/thawing cycles do not affect macrophage viability and RBCs’ membrane integrity at a concentration of up to 50 particles per cell. A small decrease for both parameters was found for the application of 5.5 µm capsules at concentrations of 10 capsules per cell and higher, which points to the advantage of the capsules of 2.7 µm and a smaller average diameter. Additionally, the circulation duration of capsules shortens with the enlargement of their size, which also reflects the advantage of small capsules. However, the first data about the efficiency of the magnetic trapping of capsules from the flow at a 30 μL/min flow rate showed a tendency of more efficient capture with the increase in capsule size and the number of freezing/thawing cycles [11].

## Error in Figure

There was a typo in **Figure 3**. “The efficiency of magnetic capture of differently sized polyelectrolyte microcapsules from the flow depending on the amount of loaded magnetite and the flow velocity.” as published. **We used the wrong name for the flow velocity units in Figure 3. It should be presented in mm/s instead of μm/s.** The **corrected** **Figure 3** appears below.

The authors state that the scientific conclusions are unaffected. This correction was approved by the Academic Editor. The original publication has also been updated.

## Figures and Tables

**Figure 3:**
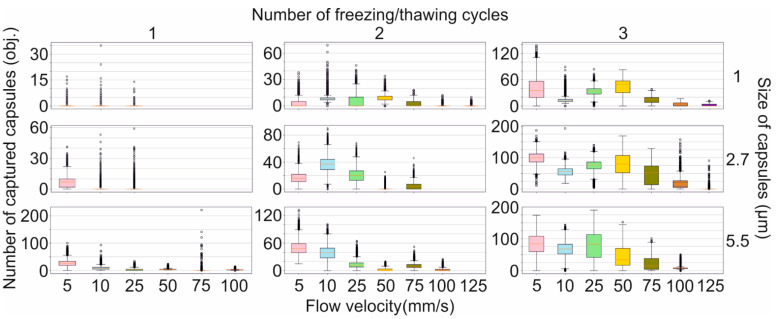
The efficiency of magnetic capture of differently sized polyelectrolyte microcapsules from the flow depending on the amount of loaded magnetite and the flow velocity.

## References

[1] Verkhovskii R., Ermakov A., Grishin O., Makarkin M.A., Kozhevnikov I., Makhortov M., Kozlova A., Salem S., Tuchin V., Bratashov D. (2022). The Influence of Magnetic Composite Capsule Structure and Size on Their Trapping Efficiency in the Flow. Molecules.

